# Integration of signaling pathway and bromodomain and extra-terminal domain inhibition for the treatment of mutant Kirsten rat sarcoma viral oncogene homolog cancer

**DOI:** 10.37349/etat.2023.00178

**Published:** 2023-10-26

**Authors:** Gerhard Hamilton, Sandra Stickler, Barbara Rath

**Affiliations:** Istituto Nazionale Tumori-IRCCS-Fondazione G. Pascale, Italy; Department of Pharmacology, Medical University of Vienna, A-1090 Vienna, Austria

**Keywords:** Non-small cell lung cancer, Kirsten rat sarcoma viral oncogene homolog, son of sevenless 1, bromodomain and extra-terminal inhibitors, proteolysis-targeting chimeras

## Abstract

Mutant Kirsten rat sarcoma viral oncogene homolog (KRAS) is now a drugable oncogenic driver and the KRAS G12C variant responds clinically to sotorasib and adagrasib that covalently block the cysteine of the active center and inhibit downstream signaling and proliferation. Unfortunately, progression-free survival (PFS) of lung cancer patients is only 5–6 months and no survival advantage has been found for sotorasib in comparison to docetaxel chemotherapy. Increased responses to KRAS inhibitors are tested in combination with the son of sevenless 1 (SOS1) inhibitors, upstream and downstream signaling modulators as well as chemotherapeutics. Some of these approaches are limited by toxicity to normal tissues and by diverse mechanisms of resistance. In essence, most of these attempts are directed to the inhibition of proliferation by impairment of the signal transduction pathways. The final target of KRAS-mediated growth stimulation is MYC in the cell nucleus that stimulates transcription of a host of genes. In detail, MYC alters genomic enhancer and super-enhancers of transcription that are frequently deregulated in cancer. Such enhancers can be targeted by bromodomain and extra-terminal (BET) inhibitors (BETi) or degraders and this review discusses whether integrated SOS1 inhibition and BET targeting of MYC synergizes against mutant KRAS tumor growth. BET degraders in the form of proteolysis-targeting chimeras (PROTACs) combined with BAY-293-mediated SOS1 inhibition revealed marked cytotoxic synergy against mutant KRAS cancer cells and may constitute a promising option for clinical treatment.

## Introduction

Kirsten rat sarcoma viral oncogene homolog (KRAS) is the most frequently mutated oncogene in human cancer and, in particular, in non-small cell lung cancer (NSCLC) that is associated with a poor prognosis. KRAS mutations result in constitutive activation of downstream signaling pathways, including v-raf murine sarcoma viral oncogene homolog B1 (BRAF), mitogen-activated protein kinase (MAPK) and the phosphatidylinositol 3-kinase (PI3K)- protein kinase B (AKT) pathway, regulating proliferation, differentiation, migration and survival [1]. Specific inhibitors have been shown to provide significant clinical benefit in pretreated NSCLC patients carrying a KRAS G12C mutation [2]. Ostrem et al*.* [3] have discovered a new allosteric KRAS pocket termed the switch-II pocket (S-IIP), which was only accessible in the guanosine diphosphate (GDP)-bound state and binds the cysteine-directed covalent inhibitors (Ostrem compounds 6, 9 and 12). Further optimization by the Wellcome Biosciences group resulted in a series of compounds culminating in ARS-1620 suited for drug administration due to efficacy and stability [4]. Further improvement of the ARS-1620 scaffold led to the first clinical KRAS G12C inhibitor AMG-510. In particular, sotorasib (AMG-510) is the first KRAS G12C inhibitor to receive approval based on significant clinical activity and a tolerable safety profile in pretreated NSCLC [5]. A similar drug, adagrasib (MRTX-849), another covalent inhibitor of KRAS G12C, has also shown efficacy in pretreated KRAS G12C patients [5]. Like other oncogene-directed therapies, intrinsic and acquired chemoresistance limits the efficacy of both agents. Trials testing KRAS inhibitors in combination with targeted agents and immunotherapy are currently ongoing to further improve clinical outcomes. KRAS mutations consist mainly of single-base mutations clustering at codon 12, 13 and 61 [6]. In lung cancer, KRAS-activating mutations are the oncogenic driver in 20–30% of cases with the dominant G12C substitution, whereas G12D is the most common mutation in pancreatic and colorectal cancer (CRC) [7]. KRAS proteins alternate between the guanosine triphosphate (GTP)-bound active state and the GDP-bound inactive state. Activation occurs by guanine exchange factors (GEFs), such as the son of sevenless 1 (SOS1), that catalyze the loading of KRAS by GTP [8]. When membrane receptor tyrosine kinases (RTKs), such as the epidermal growth factor (EGF) receptor, are activated, it recruits adapter proteins such growth factor receptor-bound protein 2 (GRB2) and src-homology 2-containing protein tyrosine phosphatase 2 (SHP2) that in turn binds SOS1, containing the RAS GEF domain. Inhibitors of these adaptor proteins, SOS1 and downstream cell signaling transmitters are tested in combination with KRAS G12C inhibitors to increase clinical responses. Another potential target to stop the KRAS-triggered malignant proliferation is the final nuclear target MYC. The MYC transcription factor controls a large panel of genes, involved in cell proliferation and survival, and its expression and stability are regulated by KRAS-triggered downstream phosphorylation [9].

## Clinical efficacy of the KRAS G12C-targeting drugs

Sotorasib covalently locks KRAS in its inactive GDP-bound state and inhibits oncogenic signaling. In the phase I/II CodeBreaK 100 clinical study 81% of the 126 patients have been pretreated with platinum-based chemotherapy and a programmed cell death protein 1/programmed cell death ligand 1 (PD-1/PD-L1) inhibitor [10]. An objective response rate (ORR) has been observed in 46 patients (37.1%), including 4 (3.2%) with a complete response (CR) and 42 (33.9%) with a partial response (PR) [8]. Disease control has been shown in 80.6% of patients and the median duration of response (DOR) was 11.1 months. The median PFS and overall survival (OS) were 6.8 months and 12.5 months, respectively. Adverse events occurred in 69.8% of patients, including 19.8% and 0.8% of grade 3 and 4 events. The 2-year follow-up of the CodeBreaK 100 trial demostrated lasting responses with sotorasib, with a 2-year OS of 32.5%. The efficacy of sotorasib (960 mg once daily) has been compared to intravenous docetaxel (75 mg/m^2^ once every 3 weeks) in an open-label manner [11]. A population of 345 patients were randomly assigned to receive either sotorasib (*n* = 171) or docetaxel (*n* = 174). A significant increase in the PFS has been detected for sotorasib compared to docetaxel (5–6 months *vs.* 4–5 months; hazard ratio 0.66). Sotorasib had a more favorable safety profile compared with docetaxel but did not prolong survival. However, in case of this CodeBreaK 200 trial, the treatment of the controls was inferior to the best standard of care and differences in the censored cases made PFS estimates unreliable and assessment of OS impossible due to relatively low patient numbers [12].

Adagrasib is similar to sotorasib but has been designed for better pharmacokinetic properties, including improved oral bioavailability, prolonged half-life, extensive tissue distribution and blood-brain barrier crossing. In the phase 2 of the KRYSTAL-1 trial, a total of 116 patients has been included, of whom almost all had been pretreated with chemotherapy and immunotherapy [13]. The ORR was 42.9%, with a median DOR of 8.5 months and median PFS and OS of 6.5 months and 12.6 months, respectively. The estimated 1-year OS was 50.8% and adagrasib has shown activity against brain metastases [8]. Although the inhibition of mutant KRAS G12C is a tremendous achievement, the responses are restricted to a subgroup of patients and are of limited duration. Novel approaches are required to improve the outcomes of mutant KRAS patients.

Combining KRAS G12C inhibitors with drugs targeting upstream or downstream signaling pathways could offer the potential to maximize therapeutic efficacy and to overcome the development of resistance. SOS1 inhibitors block the activation of KRAS and, for example, a synergistic effect of the SOS1 inhibitor BAY-293 with the KRAS inhibitor ARS-853 has been observed [14]. A phase I trial of the pan-KRAS SOS1 inhibitor BI-1701963 in patients with KRAS-mutated solid tumors is currently ongoing. Co-targeting of mitogen-activated extracellular signal-regulated kinase (MEK) and AKT signaling with KRAS-directed drugs could be an important therapeutic strategy but toxicity to normal tissues limits this approach. A negative feedback loop has been shown from extracellular signal-related kinases 1 and 2 (ERK1/2) via dual-specificity phosphatase (DUSP) and sprouty (Spry) proteins, pointing to KRAS-dependent super-enhancers [15]. Numerous trials currently test various combinations of KRAS inhibitors with signal transduction and SOS1 inhibitors, chemotherapeutics and other agents.

## Resistance to KRAS G12C inhibition

For the KRAS G12C inhibitors tumor responses are short-lived with only 5–6 months due to emerging chemoresistance to monotherapy [8, 10]. The redundancy of KRAS signaling cascades seems responsible for the secondary resistance to KRAS blockade [16, 17]. In addition to on-target mutations, acquired KRAS alterations include the upregulation of wildtype and mutant KRAS, partially by compensatory reactivation of RTKs and SOS1 [18]. Utilization of KRAS- and signaling pathway-directed combinations requires evaluation of the efficacy and the expected tolerable toxicity. The heterogeneity of the mechanisms leading to KRAS inhibitor resistance has been not fully resolved and, in addition to KRAS mutations, include cell type transformations, alternate signaling pathways activation, activating mutations in other oncogenes and more.

## Targeting KRAS by SOS1 inhibitors

Targeting all kinds of mutant KRAS alleles may be done by indirect inhibition of effectors such as the GEF SOS1 or of SHP2, both involved in the activation of KRAS [19, 20]. SHP2 is a phosphatase with diverse cellular activities, including the activation of RAS via unknown mechanisms. The small-molecule SOS1 inhibitor BI-3406 abolishes SOS1-KRAS interaction *in vitro* and in KRAS-dependent cancer models without affecting KRAS wild-type cells [21]. Furthermore, BI-3406 in combination with the MEK inhibitor trametinib prevents MAPK feedback reactivation. The SOS1 inhibitor BI-1701963 is the first to reach clinical trials for KRAS-mutated advanced solid tumors. First clinical data from a dose escalation trial of BI-1701963 revealed stable disease in 7/31 patients and good tolerance [22].

Other SOS1 inhibitors, such as the Bayer BAY-293 and the RM-0331 compounds (Revolution Medicines) target the same binding pocket as BI-3406 but has been either not optimized for clinical application or are in initial trials [23]. A range of further SOS1 inhibitors is under development, including RMC-5845, Schrödinger SDGR5, MRTX0902 from Mirati Therapeutics among others [24, 25]. SOS1 inhibitors hold promise in the combination setting, especially for inhibitors that bind KRAS in the GDP-bound state as induced by inhibition of SOS1 activity.

More efficient inhibition of KRAS-triggered signaling cascades is expected from the development of specific degraders. Proteolysis-targeting chimeras (PROTACs) specifically remove proteins of interest (POIs) via the proteasomal degradation machinery and exert stronger effects compared to merely inhibition of the target proteins [26]. These bifunctional compounds bind to a POI and form a ternary complex with an E3 ligase, resulting in ubiquitination of the target and subsequent proteolytic degradation. Boehringer Ingelheim reported a potent KRAS PROTACs BI-KRASdegrader1 that can remove all major KRAS mutants while sparing neuroblastoma RAS viral oncogene homolog (NRAS) and Harvey rat sarcoma viral oncogene homolog (HRAS) [25]. A first SOS1 PROTAC, termed 9d has been designed by linking a von Hippel-Lindau (VHL) ligand to a SOS1 agonist. 9d induced SOS1 degradation in various KRAS-mutated cancer cells and displayed higher antiproliferation activity compared to the parent SOS1 agonist [27]. These pan-KRAS drugs would be suited for non-KRAS G12C tumors such as those predominant in pancreatic ductal adenocarcinoma (PDAC).

KRAS-mutated cell lines show metabolic alterations in the form of the Warburg effect, with higher rates of glucose consumption, glycolysis and lactate production. A range of compounds for putative synergism with the SOS1 inhibitor BAY-293 using lung and pancreatic cancer cell lines, respectively, have been tested at our institution [28, 29]. Modulators of glucose utilization, such as 2-deoxyglucose (2-DG) and metformin showed synergism with BAY-293 and several chemotherapeutics, such as cisplatin, doxorubicin and topotecan, as well as MEK inhibitors, cyclin-dependent kinase (CDK) inhibitors (CDKi) and bromodomain and extra-terminal (BET) inhibitors (BETi) were found to enhance the cytotoxicity of BAY-293. Thus, not only inhibitors of KRAS downstream signaling pathways but diverse anticancer agents may be used to increase the efficacy of SOS1-directed therapy.

## KRAS: concurrent mutations

KRAS-mutant NSCLC is a heterogeneous disease of molecular subgroups bearing co-occurring genomic alterations falling mainly into three divisions comprising TP53, STK11/liver kinase B1 (LKB1) and CDKN2A/B alterations with different impacts on prognosis [30]. TP53 mutations are correlated with improved immune response due to higher expression of PD-L1 and prolonged OS. The presence of STK11 and kelch-like ECH-associated protein 1 (KEAP1) mutations in patients are associated with a shorter OS [31]. Especially, SMARCA4 and STK11 alterations are associated with worse prognosis. In detail, concurrent mutations in mutant KRAS NSCLC were identified in TP53 (38.7%), STK11 (11.8%), KEAP1 (8.1%), and CDKN2A (5.4%) [32]. Significant mutations were also detected in KRAS downstream effectors, including PIK3CA (3.8%), BRAF (2.7%) and AKT1 (1.6%). In conclusion, an efficient combination therapy would target mutant KRAS and concurrently one or several of the commutated oncoproteins.

## MYC as KRAS downstream target

MYC is universally overexpressed in rapidly proliferating cells [33]. MYC indirectly acts as a global transcription amplifier via interactions with acetyltransferases p300, CREB binding protein (CBP) and the WD repeat-containing protein 5 (WDR5) histone lysine methyltransferase complex [34]. MYC protein is found at low levels in normal cells under control of the ubiquitin-proteasome pathway. Target genes include cyclins D1, D2, B1 and CDK4 and MYC also interferes with the function of p21 and p27 inhibitors of CDKs. MYC must first heterodimerize with MAX to become transcriptionally active [35]. MYC also releases a paused elongation of transcription through the elongation factor and kinase positive transcription elongation factor b (P-TEFb). Interaction of MYC with additional cofactors such as WDR5, RNA polymerase II-associated factor 1 (PAF1) and bromodomain protein bromodomain-containing protein 4 (BRD4) are likely to direct MYC to certain chromatin sites [35]. High levels of these different factors are supposed to generate super-enhancers that cause collective target gene expression.

Amplification of MYC is one of the most common genetic alterations in lung cancer, affecting growth, invasion and drug resistance [36]. MYC controls the expression of approximately 30% of the human genes and its overexpression is aberrantly in cancer cells [37, 38]. MYC represents an effector of multiple signaling pathways as the MAPK and the PI3K pathways converge in activation of the nuclear MYC protein at the post-translational level [39]. Both signaling cascades synergistically enhance MYC protein stability by phosphorylation in the transactivation domain, leading to MYC accumulation [40].

Upon ligand binding, membrane RTKs recruit an intracellular complex of GRB2, SHP2 and SOS1 that stimulate the GTP loading of KRAS to an active state. Downstream signaling cascades include the RAF-MEK-ERK1/2 MAPK and the PI3K-AKT-mTOR pathways eventually converging in the stabilization of MYC in the cell nucleus. In connection with BET protein BRD4, P-TEFb and WDR5 the MYC/MAX protein complex activates the transcription of a host of target genes. BETi block MYC directly or via the bromodomain protein BRD4 and other family members. In contrast to the classical BETi JQ1, the PROTACs MZ1, dBET57 and ARV-771 remove the BET proteins via ubiquitination and proteasomal degradation in multiple cycles.

Small-molecule inhibitors of MYC are in preclinical development but as an alternative way of modulation, BET bromodomain inhibition suppresses MYC expression in hematologic malignancies [41]. MYC is an intrinsically disordered protein (IDP) and lacks the requisite deep hydrophobic pockets for inhibitor binding. The gain of function of MYC in cancer cells results from overexpression or from amplification but rarely from mutation [42]. Phosphorylation at serine 62 by MAPKs acts as a downstream signal of oncogenic KRAS or CDKs and influences activity, target selection and intracellular localization of MYC. The growth of mutant KRAS tumors is dependent on the function of MYC and, thus, targeting KRAS-driven NSCLC cells with BET inhibition appears feasible. The classical BETi JQ1 is active in NSCLC cells but its activity in mutant KRAS cells is abrogated by other alterations or downregulation of LKB1*.* MYC inhibition had little to no side effects in normal tissue [35].

**Figure 1:**
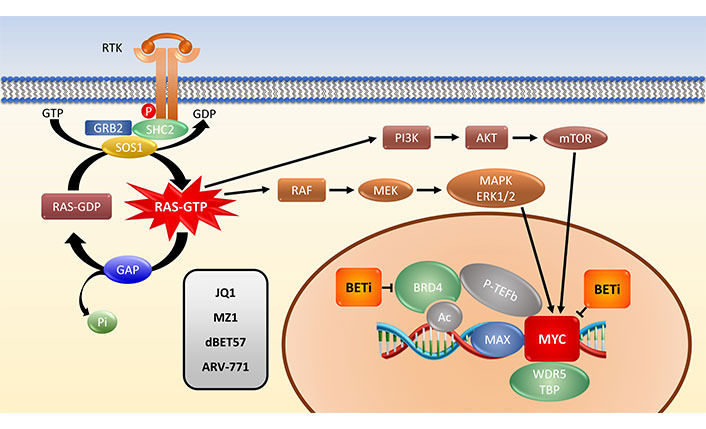
Schematic representation of the KRAS-MYC link. P: phosphate group; SHC2: SHC-transforming protein 2; GAP: GTPase-activating protein; Pi: inorganic phosphate; Ac: acetylation

Targeting/inhibition of the bromodomain protein BRD4 ultimately leads to down-regulation of the MYC protein. BETi are not restricted to *MYC* gene regulation, but many BET drugs are currently in clinical trials for MYC inhibition therapy [43]. The relationship between KRAS and MYC in tumorigenesis is very complex but BET inhibition in mouse models of KRAS-driven PDAC and NSCLC reduced both tumor size and tumor grade [44, 45]. In good accordance, significant cytotoxicity of BETi against a panel of RAS-mutated human cancer cell lines (8 PDAC and 6 NSCLC) has been demonstrated [45]. However, at least in PDAC cells its anti-tumorigenic effect is independent of MYC regulation. LKB1-wild type NSCLC cells display a significantly higher sensitivity to these inhibitors and a decrease of MYC. BRD4 is a MYC coactivator expressing a chromatin acetyl-lysine recognition domain that recruits the elongation factor p-TEFb to specific chromatin locations (Figure 1) [46]. BRD4 represents an epigenetic regulator of transcription with intrinsic kinase and histone acetyltransferase (HAT) functions. Bromodomain inhibitors, such as JQ1, lead to the inhibition of MYC transcription and to genome-wide downregulation of MYC targets [46].

## BET proteins

BRD4 is a member of the BET family comprising BRD2, BRD3, BRD4 and bromodomain testis-specific (BRDT) that recruits transcription complexes to specific locations of the DNA to initiate gene expression. In detail, acetylated lysine residues introduced by HATs are recognized by bromodomain readers, that may increase the transcription of oncogenes [47, 48]. BET proteins bind acetylated histones through two bromodomains, BD1 or BD2, and recruit the P-TEFb [49]. Consistent with the BET regulation of cell cycle genes, knockdown of BRD4 leads to arrest in S phase in some cell types [50].

Aberrant BRD4 expression may contribute to the progression of hematological malignancies and several solid cancers [51]. Oncogenic KRAS acts on the stabilization of the MYC protein via activated ERK1/2 and phosphorylation of MYC at serine 62 that prevents the proteasomal degradation [9]. The overexpression of BRD4 appears to transcriptionally activate genes involved in cell cycle progression and survival [44]. The activation of PI3K and MAPK signaling cascades appears to cause intrinsic resistance to BETi in cancer [48, 52]. In KRA*S* mutant NSCLC, sensitivity to BETi was reported, but only with high concentrations of at least 2.5 µmol/L JQ1, exceeding clinically achievable concentrations. MYC downregulation was observed at 500 nmol/L JQ1 in the absence of an anti-proliferative effect, excluding MYC downregulation as a major source [41]. The presence of a KRAS mutation has been demonstrated to be linked with the overexpression of BET proteins including BRD2, BRD3 and BRD4 in pancreatic cancer [53]. So far, no BRD inhibitor has been granted Food and Drug Administration (FDA) approval [54]. Clinical experience with BETi has been disappointing and long-lasting responses were scarce [55]. Difficulties are due to a narrow therapeutic window and to on-target adverse events, such as thrombocytopenia.

## BET PROTACs

PROTACs employ the cellular proteasomal degradation system for the removal of POIs including structural proteins. A binder of the target protein is covalently linked to an E3 ubiquitin ligase ligand that mediates ubiquitination of the POI and mark it for disposal by the proteasome [56]. After complex formation and ubiquitin transfer, the PROTAC dissociates and recycles on to the next POI, such gaining much higher efficacy compared to the corresponding inhibitor. Preclinically, a very low PROTAC dose can profoundly reduce protein levels, including its function a possible scaffold. First PROTACs from Arvinas (New Haven, CT, USA) can be administered orally, showing adequate plasma drug concentrations and good safety in phase I along with detectable antitumor activity. ARV-471 (Arvinas, New Haven, CT, USA) yielded a clinical benefit rate of 38% in the evaluable breast cancer patients [57].

In detail, PROTACs has attracted considerable attention owing to their great potential for use in cancer treatment, especially to target structural proteins [56, 58, 59]. Transcription factors regulate gene transcription and thus constitute important targets to interfere with malignant growth. A host of small-molecule inhibitors (SMIs), such as JQ1, OTX15, BI-894999, CPI-0610 and others, have been shown to target BET proteins but corresponding PROTACs, such as MZ1, dBET57 and ARV-771, show much higher activity and inhibit oncogene expression [60–62]. The ARV-771 PROTAC, a small-molecule pan-BET degrader has improved efficacy in models of castrate-resistant prostate cancer (CRPC) as compared with BET inhibition [63]. ARV-771 is a VHL E3 ligase-based BET PROTAC that achieves rapid BET protein degradation with DC_50_ (the PROTAC concentration that results in 50% degradation) values < 1 nmol/L. ARV-771 also has an antiproliferative effect that is up to 500-fold more potent than the BETi JQ1 and OTX015.

In general, PROTACs have high molecular weights and the resulting low permeability and solubility may hinder cellular uptake and target degradation, as well as a considerable active transporter-mediated efflux [64]. The development of orally available clinically grade PROTACs requires extensive screening and optimization with respect to bioavailability, antitumor activity and pharmacological properties. PROTACs degrade their POI in both tumor and normal cells, meaning that the therapeutic and toxic targets are the same [58].

## Synergy of SOS1 inhibition and BET degradation in KRAS mutant cells

The protumor mechanisms linking mutant KRAS-BETs and MYC offer the possibility to impair the effects of MYC on gene expression by BET-targeting agents, preferably in form of PROTACs with high activity and achievable at low *in vivo* concentrations. The putative interaction of the SOS1 inhibitor BAY-293 (Merck, Darmstadt, Germany) and the BET PROTAC ARV-771 (Arvinas, New Haven, CT, USA) has been checked using BH1467 (KRAS G12C NSCLC) and EGI-1 (KRAS G12A, cholangiocarcinoma cell line) in pilot experiments at our institution. BH1467 is a proprietary NSCLC established in at our institution from the pleural effusion of a patient progressing under sotorasib therapy. EGI-1 was acquired from the German Collection of Microorganisms and Cell Cultures (DSMZ, Braunschweig, Germany). Both cell lines were kept in Roswell Park Memorial Institute (RPMI)-1640/10% FBS under cell culture conditions.

**Figure 2:**
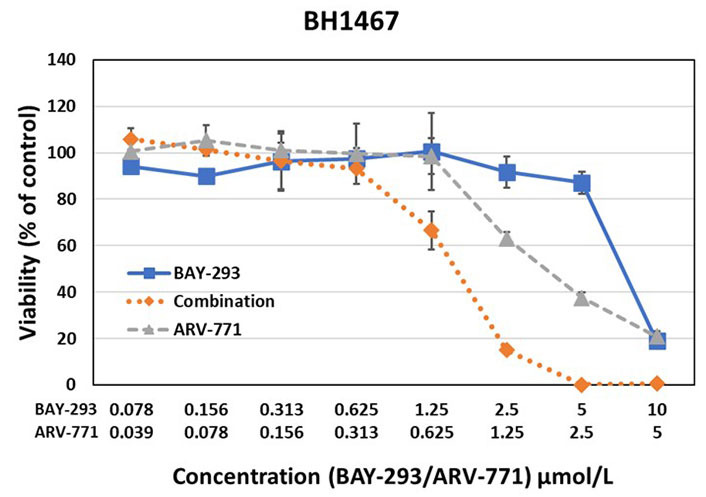
Testing of the synergistic activity of the SOS1 inhibitor BAY-293 and the BET PROTAC ARV-771 against the BH1467 KRAS mutated NSCLC cell line yielded a CI of 0.63, indicating high synergism

**Figure 3:**
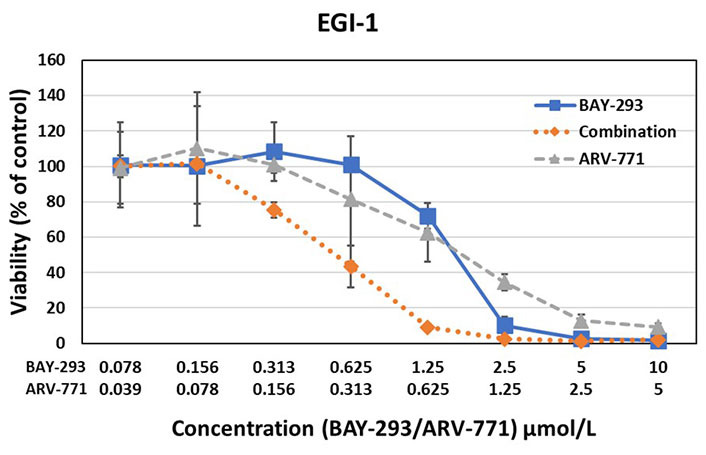
Testing of the synergistic activity of the SOS1 inhibitor BAY-293 and the BET PROTAC ARV-771 against the EGI-1 KRAS mutated cholangiocarcinoma cell line yielded a CI of 0.44, again indicating high synergism

The effects of BAY-293 and ARV-771 were tested alone and in combination in preliminary cell cytotoxicity assays as shown for BH1467 in Figure 2, and for EGI-1 cells in Figure 3. The combination test employs both drugs at the concentrations used for single use. For this dose-response curves, the drugs were diluted in 7 twofold dilution steps. The combinations were evaluated according to the Chou-Talalay method, demonstrating drug synergy by a combination index (CI) below 1.

The results of the BAY-293-ARV-771 tests exhibited marked synergy between this SOS1 inhibitor and a BET PROTAC. This pilot experiment documented synergy of the inhibitors in two KRAS mutated cells with a completely differing NSCLC and cholangiocarcinoma cellular background.

## Conclusions

The KRAS G12C inhibitors yield a limited clinical response and the other KRAS alleles are not tackled clinically so far. Superior outcomes are thought to be achieved by combinations of KRAS/SOS1 inhibitors with upstream or downstream signaling mediators. However, this approach is limited by resistance and the off-target toxicity to normal tissues and, therefore, may achieve a gradual improvement at best. The downstream target of KRAS, namely MYC, is currently difficult to target by small-molecule drugs. However, the integration of KRAS-driven signaling and inhibition of MYC and other protumor effectors with the inhibition or degradation of BET proteins seem to provide a much more efficient way of inhibition. A wide range of KRAS and SOS1 inhibitors are available and, recently, oral available PROTACs suitable for clinical application has been successfully developed. The examples shown for the combinations of the SOS1 inhibitor BAY-293 and the bromodomain protein-directed PROTAC ARV-771 demonstrate the markedly synergism of such a drug combination. Additionally, the PROTACs can be finetuned by combining different binders, distinct linkers and the wide range of E3 ligase reactive groups with the potential to incorporate tissue specific components. These new drugs seem to be effective in very low concentrations exceeding the activity of BETi by decades due to degradation of POIs and recycling of the agents. As special advantage, the BET degraders are expected to remove other oncogenes regulated by super-enhancers that are co-mutated in cancer and influence outcomes. This combination reveals promising activity for a future clinical application.

## Abbreviations

AKT: protein kinase B
ARV: Arvinas
BET: bromodomain and extra-terminal
BETi: bromodomain and extra-terminal inhibitors
BRD4: bromodomain-containing protein 4
CDK: cyclin-dependent kinase
CI: combination index
ERK1/2: extracellular signal-related kinases 1 and 2
GDP: guanosine diphosphate
GEFs: guanine exchange factors
GTP: guanosine triphosphate
KRAS: Kirsten rat sarcoma viral oncogene homolog
LKB1: liver kinase B1
MAPK: mitogen-activated protein kinase
MEK: mitogen-activated extracellular signal-regulated kinase
NSCLC: non-small cell lung cancer
OS: overall survival
PDAC: pancreatic ductal adenocarcinoma
PFS: progression-free survival
PI3K: phosphatidylinositol 3-kinase
POIs: proteins of interest
PROTACs: proteolysis-targeting chimeras
P-TEFb: positive transcription elongation factor b
RTKs: receptor tyrosine kinases
SHP2: src-homology 2-containing protein tyrosine phosphatase 2
SOS1: son of sevenless 1
WDR5: WD repeat-containing protein 5

## Declarations

### Copyright

© The Author(s) 2023.
